# Statins Use in Alzheimer Disease: Bane or Boon from Frantic Search and Narrative Review

**DOI:** 10.3390/brainsci12101290

**Published:** 2022-09-24

**Authors:** Nawal Alsubaie, Hayder M. Al-kuraishy, Ali I. Al-Gareeb, Bandar Alharbi, Michel De Waard, Jean-Marc Sabatier, Hebatallah M. Saad, Gaber El-Saber Batiha

**Affiliations:** 1 Department of Pharmacy Practice, College of Pharmacy, Princess Nourah bint Abdulrahman University, Riyadh 11564, Saudi Arabia; 2 Department of Pharmacology, Toxicology and Medicine, Medical Faculty, College of Medicine, Al-Mustansiriyah University, Baghdad P.O. Box 14132, Iraq; 3 Department of Pharmaceutical Services, Prince Sultan Military Medical City, Riyadh 12233, Saudi Arabia; 4 Smartox Biotechnology, 6 rue des Platanes, 38120 Saint-Egrève, France; 5 L’institut du Thorax, INSERM, CNRS, Université de Nantes, 44007 Nantes, France; 6 LabEx «Ion Channels, Science & Therapeutics», Université de Nice Sophia-Antipolis, 06560 Valbonne, France; 7 Institut de Neurophysiopathologie (INP), CNRS UMR 7051, Faculté des Sciences Médicales et Paramédicales, Aix-Marseille Université, 27 Bd Jean Moulin, 13005 Marseille, France; 8 Department of Pathology, Faculty of Veterinary Medicine, Matrouh University, Mersa Matruh 51744, Egypt; 9 Department of Pharmacology and Therapeutics, Faculty of Veterinary Medicine, Damanhour University, Damanhour 22511, Egypt

**Keywords:** Alzheimer’s disease, statins, cognitive functions

## Abstract

Alzheimer’s disease (AD) was used to describe pre-senile dementia to differentiate it from senile dementia, which develops in the adult age group of more than 65 years. AD is characterized by the deposition of amyloid beta (Aβ) plaque and tau-neurofibrillary tangles (TNTs) in the brain. The neuropathological changes in AD are related to the deposition of amyloid plaques, neurofibrillary tangles, and progression of neuroinflammation, neuronal mitochondrial dysfunction, autophagy dysfunction, and cholinergic synaptic dysfunction. Statins are one of the main cornerstone drugs for the management of cardiovascular disorders regardless of dyslipidemia status. Increasing the use of statins, mainly in the elderly groups for primary and secondary prevention of cardiovascular diseases, may affect their cognitive functions. Extensive and prolonged use of statins may affect cognitive functions in healthy subjects and dementia patients. Statins-induced cognitive impairments in both patients and health providers had been reported according to the post-marketing survey. This survey depends mainly on sporadic cases, and no cognitive measures were used. Evidence from prospective and observational studies gives no robust conclusion regarding the beneficial or detrimental effects of statins on cognitive functions in AD patients. Therefore, this study is a narrative review aimed with evidences to the beneficial, detrimental, and neutral effects of statins on AD.

## 1. Introduction

Dementia is regarded as a clinical syndrome characterized by cognitive dysfunction [1]. Alzheimer’s disease (AD) is the most common type of dementia. AD was firstly described by Alois Alzheimer in 1907 in women who presented with memory deficit and neuropsychiatric disorders [1]. Over time, AD was used to describe pre-senile dementia to differentiate it from senile dementia [2]. However, AD is not bimodal of onset as AD is identified as a single neurological entity; its prevalence is sharply increased after the age of 65 [3]. Thus, AD must be distinguished from other types of dementia including reversible dementia, Parkinson-associated dementia, vascular dementia, and frontotemporal dementia. Nevertheless, till now, there is no specific biomarker that differentiates AD from other types of dementia. As well, brain biopsy and brain positron emission tomography were used in the diagnosis of AD but with conflicting findings [4]. AD represents 70% of all dementia types; it upsurges with age, doubling every 10 years. It has been reported that AD prevalence is 3% in individuals aged 65–74, to about 50% in subjects aged 85 years and older [5]. In the United States, the prevalence of AD was about 5 million in 2007 that was suspected to increase to 13 million in 2050 [6]. Moreover, head trauma, female sex, low education level, cardio-metabolic disorders, and vascular diseases are regarded as risk factors for the development of AD [7].

**Figure 1:**
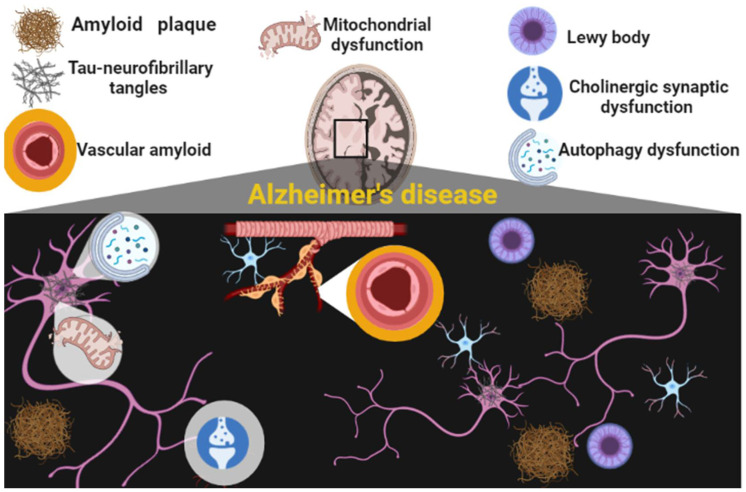
Pathophysiology of Alzheimer’s disease (AD): Amyloid plaques, mitochondrial dysfunction, formation of tau-neurofibrillary tangles, vascular amyloid, autophagy dysfunction, and cholinergic dysfunction are linked with AD neuropathology.

The pathological distinctive features of AD are senile and neuritic plaques with noteworthy dendritic loss, dystrophic neuritis, neuropil threads, and cerebrovascular amyloid [8]. Though, synaptic loss, amyloid pathway, and Lewy body deposition are the chief pathological feature of AD [8,9]. In addition, AD is characterized by the deposition of amyloid beta (Aβ) plaque and tau-neurofibrillary tangles (TNTs) in the brain. Notably, deposition of Aβ occurs earlier than the pathology of cortical tau. Deposition of Aβ-induces tau-mediated neuronal and synaptic loss in AD, though tau neuropathology can advance independently of Aβ accumulation [8,9]. The neuropathological changes in AD are related to the deposition of amyloid plaques, neurofibrillary tangles, and progression of neuroinflammation, neuronal mitochondrial dysfunction, autophagy dysfunction, and cholinergic synaptic dysfunction (Figure 1).

Moreover, some investigations and research studies suggest alternative mechanisms that drive AD or act in combination with Aβ to promote neurodegeneration development [10]. For example, blood brain barrier (BBB) dysfunction may develop prior to Aβ deposition mainly in patients positive for ApoE [10]. As well, endothelial dysfunction and imbalance in the production of growth factors and nitric oxide (NO) could be potential mechanisms for development of AD neuropathology [10].

Interestingly, MicroRNAs (miRNAs) are non-coding 19–25 nucleotides long, single-stranded protein molecules that are endogenously synthesized and evolutionarily conserved [11,12]. The role of miRNAs is imperative in several cellular processes, such as cellular proliferation, cell differentiation, apoptosis, and in neural cell biology. Local protein synthesis is crucial to the maintenance of neuronal function and synaptic plasticity. Several hundreds of translationally silent mRNAs exist in axons; their activation via stimuli leads to their subsequent protein synthesis specific to that cellular process. miRNAs are located in cell soma, dendrites, and synaptosomes; they control AMPA-type glutamate receptor (AMPAR) along with GABA receptor A (GABA_A_Rs), crucial for synaptic coordination and long-term potentiation [13]. Hence, miRNAs are regarded as key regulators of local protein synthesis during synaptic plasticity. miRNAs are implicated in several pathological processes, such as cancer, diabetes, and neurodegenerative diseases. The role of miRNAs in regulating endoplasmic reticulum (ER) stress is the foundation to unravel, following several studies. Several miRNA families, for instance miR-485, were found to be dysregulated in AD and other neurological diseases [11,12,13]. Notably, miRNAs exhibit a crucial role in the pathogenesis of several neurodegenerative diseases, such as AD; they may be either upregulated or downregulated in AD. The onset of AD begins with synaptic dysfunction, which is accompanied by the dysregulation of several miRNAs. miRNAs serve as a promising biomarker to monitor initial, pre-symptomatic phases of AD pathogenesis. Evidence from previous studies suggests that several miRNAs are implicated in the AD pathogenesis by dysregulating functions of Aβ production, Cofilin, APP, BACE1, and Tau phosphorylation levels [14]. Chen et al. suggests that using miR-331-3p and miR-9-5p, along with autophagic activity and Aβ plaques, may distinguish early versus late stage of AD for more accurate and timely diagnosis. Additionally, we further provide a possible new therapeutic strategy for AD patients by inhibiting miR-331-3p and miR-9-5p and enhancing autophagy [14]. Notably, miRs have been proposed to mediate the pleiotropic effects of statins including immunomodulatory and anti-inflammatory effects [15]. miRNAs are implicated in statin-related interindividual variations in therapeutic response, directly via HMG-CoA reductase, or indirectly through targeting cytochrome P450 3A (CYP3A) functionality and proprotein convertase subtilisin/kexin type9 (PCSK9) biology [15]. Therefore, upregulation of miRNAs by statins may affect AD pathophysiology.

**Figure 2:**
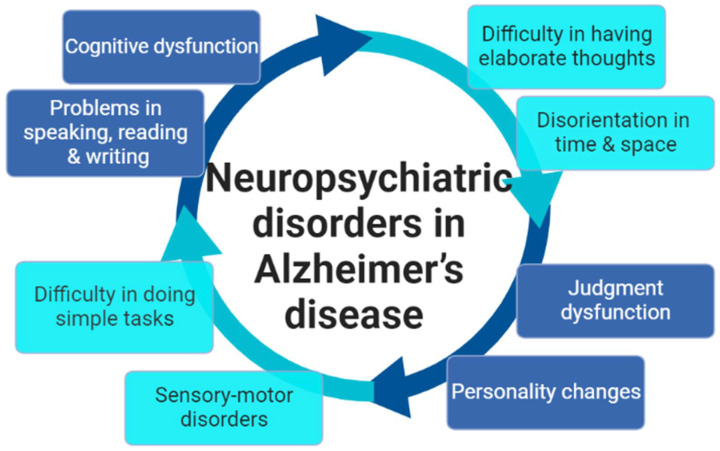
Neuropsychiatric disorders in Alzheimer’s disease (AD): Neuropsychiatric disorders in AD are interrelated disorders ranging from personality changes to severe cognitive dysfunction.

The clinical presentation of AD patients is characterized by memory loss, cognitive dysfunction, speech dysfunction, sensory-motor disorders, and personality changes. Despite these features, some AD patients remain stable for several years and may improve with time [16]. Additionally, early-onset AD is more progressive and characterized by less memory loss with higher psychosocial disorders [16,17]. Genuine AD patients presented with apraxia, judgment dysfunction, speech abnormalities, disorientation, and personality changes [16,17]. The net clinical findings in AD patients are mainly neuropsychiatric disorders (Figure 2).

Other types of dementia may contribute to AD-related cognitive decline such as vascular dementia (VaD), which is often observed with AD. VaD is a heterogeneous disease genetically related to cerebrovascular disease [18]. VaD is the second most cause of dementia, characterized by cerebral infarcts, white matter lesions, myelin loss, and often amyloid angiopathy. Hence, vascular damage is a critical cause of neuronal loss and synaptic disintegration. Abnormal neuroinflammation, autophagy, and apoptosis are the prerequisite factors for endothelial and neuronal cell damage. This leads to the onset and progression of cerebrovascular disorders and cognitive dysfunction [19]. The breakdown of neurovascular coupling is evident across a wide variety of both neurological and psychiatric disorders including AD [19]. Pathological changes to the neurovascular unit and endothelia impair the signaling pathways involved in neurovascular coupling, leading to brain pathologies ranging from subtle cognitive deficits to severe AD [20].

**Figure 3:**
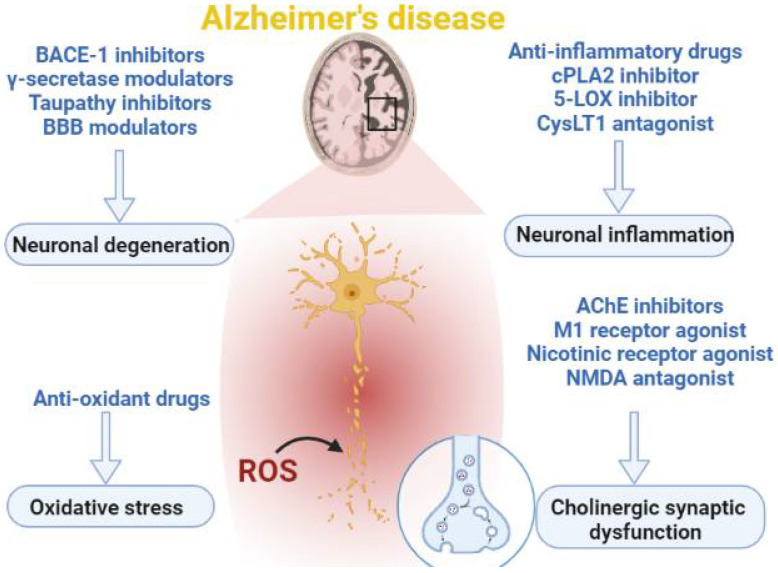
Drugs affecting the pathogenesis of Alzheimer’s disease (AD): Different modalities of drugs used in the management of AD. Anti-inflammatory, which reduces neuronal inflammation; γ secretase inhibitors, which attenuate neuronal degeneration; acetylcholine esterase (AChE) inhibitors, which prevent cholinergic synaptic dysfunction; and antioxidants, which prevent oxidative stress-induced neuronal degeneration in AD.

Management of AD patients is mainly symptomatic relief by cholinesterase inhibitors, such as donepezil, rivastigmine, tacrine, and galantamine. As well, N-methyl-D-aspartate (NMDA) antagonists such as memantine are used in treating cognitive deficits in AD patients [21,22] (Figure 3). Nevertheless, these agents provide symptomatic relief without affecting the etiopathologic progression of AD [21,22].

Most AD patients are associated with cardiometabolic disorders and being treated with statins. Therefore, statins may affect cognitive functions in AD patients. It has been reported that statins affect cognitive functions in positive and negative fashions [23,24]. Statins improve reaction time in the old age group, whereas it reduces functional working memory in the younger age group [23].

### Aim of the Study

Based on this scientific rationale, this narrative review aimed to find the positive and negative impacts of statins on the clinical course and cognitive function of AD patients regarding the assorted view of preponderance.

## 2. Statins Overview

**Figure 4:**
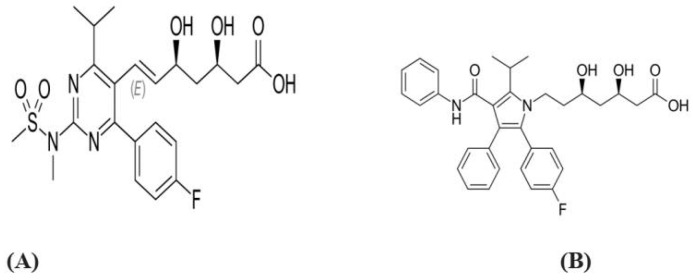
Chemical structure of statins (**A**): Rosuvastatin (**B**): Atorvastatin [26,27].

Statins such as atorvastatin and rosuvastatin (Figure 4) [25] are also called 3-hydroxy-3-methyl-glutaryl-coenzyme A (HMG-CoA) inhibitors.

Statins inhibit HMG-CoA involved in cholesterol biosynthesis; precisely, they compete with HMG-CoA in the mevalonate pathway [28]. De novo cholesterol biosynthesis in the liver mainly occurs at night, so short half-life statins such as simvastatin should be given at night, while long-acting statins such as atorvastatin and rosuvastatin can be taken at any time [29]. Decreasing circulating cholesterol induces hepatic cells to synthesize low-density lipoprotein (LDL) receptors to compensate for this pathway. The net effects of statins are the reduction in LDL and VLDL with an increment in HDL serum levels due to the modulatory effects of statins on the expression of lipoprotein receptors [30].

Moreover, statins therapy reduces the synthesis of isoprenoids, such as geranylgeranyl pyrophosphate and farnesyl pyrophosphate, with subsequent reduction in Rho-associated protein kinase [29]. Inhibition of protein prenylation is responsible for the pleiotropic effects of statins, such as immune modulation, protection of endothelial cells, and cardioprotection [29,30]. Likewise, inhibition of protein prenylation is linked with the development of adverse effects, such as myalgia, neuropathy, and hyperglycemia [25].

Pleiotropic effects of statins through different mechanisms are engaged in the use of statins for the management of endothelial dysfunction, atherosclerosis, thrombosis, and vasculitis [31,32,33]. Thus, statins therapy may indicate in different cardiovascular disorders regardless of lipid profile. In addition, statins are used for primary prevention of cardiovascular diseases in high-risk people and as secondary prevention for patients with cardiovascular complications [32,34,35,36].

There are two types of statins: either natural, such as lovastatin and mevastatin, or synthetic, such as atorvastatin, fluvastatin, cerivastatin, and rosuvastatin [33,37]. As well, fermentation-derived statins such as simvastatin and pravastatin are regarded as semisynthetic statins [38]. In addition, statins are classified according to their lipophilicity into water-soluble, such as rosuvastatin, and lipid-soluble, such as atorvastatin [34,39]. The main adverse effects of statins are rhabdomyolysis, especially when combined with fibrate lipid-lowering agents. Statins may increase the risk of new-onset type 2 diabetes mellitus (T2DM) and peripheral neuropathy via interaction with pancreatic β cells and neuronal metabolism, respectively [35,40]. Data from retrospective clinical studies suggest that statins increase the probability of inducing T2DM [20,41]. Exposure to statins significantly enhanced intracellular free fatty acid levels in skeletal muscle, which may inhibit the insulin signaling cascade to hamper glucose clearance. Free fatty acids are known to inhibit insulin signaling via the protein kinase C (PKC) pathway [42]. Cumulatively, these data suggest that in vivo statins may induce glucose intolerance by the free fatty acid mediated pathway, although other unknown mechanisms cannot be ruled out [42,43]. It has also been shown that fluvastatin, another member of the statin family of drugs, regulates insulin sensitivity in adipose tissue [44]. Moreover, alterations of gut microbiota by long-term statins therapy may induce insulin resistance (IR), hyperglycemia, and development of overt T2DM [45]. In this bargain, development of peripheral IR and brain IR may contribute to the neuropathology of AD [46]. The molecular signaling pathways through which insulin exerts its actions in the body also mediate its roles in synaptic neurotransmission, neuronal and glial metabolism, and the neuroinflammatory response in the brain [45]. Brain IR can be defined as the failure of brain cells to respond to insulin, resulting in impairments in synaptic, metabolic, and immune response functions [45,46]. T2DM is associated with brain IR, and studies suggest that brain IR is a feature of AD; however, whether the two conditions are mechanistically linked or represent unrelated occurrences in ageing is unclear [45,46,47]. Herein, statins-induced IR and T2DM could be the possible mechanistic pathway by which statins affect AD pathology.

## 3. Statins and AD

Statins are one of the main cornerstone drugs for the management of cardiovascular disorders, regardless of dyslipidemia status via their pleiotropic effects. The increasing use of statins, mainly in the elderly groups for primary and secondary preventions of cardiovascular diseases, makes them a commonly used medication globally [32,33,48]. It has been shown that 30% of people in the USA over the age of 40 are on statins [32,33,35]. Extensive and prolonged use of statins may affect the cognitive functions in healthy subjects and dementia patients [24]. Statins-induced cognitive impairments in both patients and health providers had been reported, according to the post-marketing survey. This survey depends mainly on sporadic cases, and no cognitive measures were used. However, many patients with underlying cardiovascular disorders on statins pharmacotherapy experience some aspects of cognitive impairment [49]. Evidence from prospective and observational studies gives no robust conclusion regarding the beneficial or detrimental effects of statins on the cognitive functions in AD patients [50,51,52]. Randomized controlled trials and well-conducted observational studies of baseline statin use and subsequent cognition over several years of follow-up do not support a causal preventative effect of late-life statin use on cognitive decline or dementia [51]. Similarly, statins given in late life to individuals at risk of vascular disease have no effect in preventing AD or dementia [52]. Biologically, it seems feasible that statins could prevent dementia due to their role in cholesterol reduction, and initial evidence from observational studies was very promising [52]. Notably, short-term use of statins did not affect cognitive function, while long-term pharmacotherapy of statins affects cognitive functions positively or negatively in different manners [24,53].

There are inadequate clinical data to sustain the use of statins for decreasing the risk of AD. As publicity becomes more widespread regarding the possible benefits of statins on AD, clinicians encounter patients and family history of this disease [49]. Therefore, are patients with hypercholesterolemia or normal cholesterol with cardiovascular complications treated with statins at higher risk for the development of AD, or is the reverse true? These findings need to be addressed.

The potential link between the positive–negative impacts of statins pharmacotherapy on the incidence and progression of AD remain tentative, requiring extensive research: including whether statins pharmacotherapy increases or decreases the progression of AD, whether there is an association between cholesterol level and grade of AD concerning the family history, and whether statins pharmacotherapy used in middle life and over the age of 60 affect the incidence and progression of AD or not [54]. A previous longitudinal study observed that patients with hypercholesterolemia in middle age were at high risk for the development of AD [55]. Thus, correction of cardiovascular risk factors by statins pharmacotherapy could be beneficial in the prevention of AD. However, the use of statins pharmacotherapy in patients with underlying cardiovascular complications but with normal cholesterol may associate with the development of cognitive impairment [56,57].

### 3.1. Detrimental Effects of Statins on AD

It has been reported from earlier studies that statins therapy was associated with reversible cognitive dysfunction [58]. For example, atorvastatin in a phase one clinical trial led to reversible cognitive impairment [58]. Following that, mainly in phase three, cognitive dysfunction was not reported as a potential adverse effect [50]. However, post-marketing surveillance, based on many reported cases, disclosed a potential link between statins use and the development of cognitive dysfunction in a reversible manner [59]. A randomized, double-blind clinical trial that included 308 healthy subjects for the effect of lovastatin on cognitive function showed this drug led to significant impairment of psychomotor performance and working memory compared to the placebo effect [60]. Evans et al. results from 171 patients illustrated that discontinuation of statins pharmacotherapy reversed statins-induced cognitive impairment [61]. Results from this study proposed that statins therapy has different onset and recovery courses of cognitive dysfunction. Based on these findings, in 2012, the FDA declared that doctors should inform patients about the risk of cognitive impairment before prescribing statins [62]. However, the FDA stated that the protective effects of statins on cardiovascular diseases outweigh mild cognitive impairments [62].

Furthermore, various studies were conducted to explore the association between cognitive deficit and statins use. Of note, lipophilic statins such as atorvastatin and simvastatin are associated with a higher percentage of reversible cognitive dysfunction [63,64]. Interestingly, patients with cognitive disorders are likely to be affected by the negative impact of statins on cognitive function [65]. However, hydrophilic pravastatin leads to more cognitive impairment compared to lipophilic atorvastatin in adult rats [66]. Lipophilic statins have more penetration capabilities than hydrophilic statins [67] thus; lipophilic statins may affect the brain in a robust way. A population-based, retrospective cohort study illustrated that lipophilic and fungus-derived statins are associated with a higher risk for progression of AD compared to hydrophilic statins. Additionally, statins potency does not affect the risk for AD [67]. This variation in the effect of statins needs to be verified by future studies.

The underlying mechanisms for statins-induced cognitive dysfunction are related to the reduced availability of cholesterol, which is necessary for glial and neuronal membrane integrity [68]. As well, statins-induced reduction of CoQ10 may impair neuronal mitochondrial function causing oxidative stress and neuronal injury [69]. Dumont et al. found that CoQ10 had a potential effect on the reduction of βA pathology and improve cognition in the transgenic mice model of AD [70]. Thus, treatment with CoQ10 could be effective in the management of AD by reducing oxidative stress and inflammatory changes [71,72,73].

These verdicts suggest that prolonged statins therapy, mainly in older patients, may increase the risk for the development of AD.

**Figure 5:**
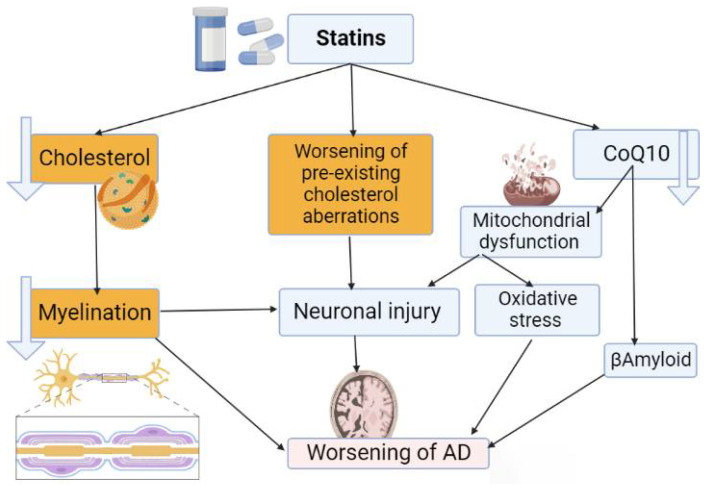
Detrimental effects of statins on Alzheimer’s disease (AD): Statins-induced AD are mediated through reduction of neuronal cholesterol with reduction of neuronal myelination. As well, statins lead to neuronal injury by inducing reduction of CoQ10 and worsening of preexisting cholesterol aberrations with induction of oxidative stress and mitochondrial dysfunction.

Excessive inhibition of brain cholesterol biosynthesis by statins, mainly the lipophilic ones, reduces neuronal myelination and development of cognitive dysfunction. Lipophilic statins produce more severe cognitive impairment due to higher suppression of brain cholesterol biosynthesis [49]. Indeed, preexistence of a cholesterol defect increases the risk of statins-induced AD [74]. The greater negative impact of lipophilic statins could be related to the induction of a pro-inflammatory microenvironment. In vitro and in vivo studies demonstrated that lipophilic statins trigger the release of pro-inflammatory cytokines from human monocytes [75]. As well, lipophilic statins induce the generation of reactive oxygen species (ROS) in monocytes [75,76]. However, hydrophilic rosuvastatin had an anti-inflammatory effect through suppression release of pro-inflammatory cytokines from cultured microglial cells [77]. Therefore, induction of pro-inflammatory cytokines and ROS by lipophilic but not by hydrophilic statins may explain the negative impact of lipophilic statins on cognition and the risk for propagation of AD (Figure 5). A preclinical study demonstrated that the use of lovastatin promotes the formation and deposition of Aβ amyloid in female mice [78]. Of note, low levels of isoprenoid and cholesterol promote the deposition of Aβ amyloid in mice [78]. However, these experimental findings cannot exclusively correlate with clinical findings in AD patients. Of interest, Bagheri et al., 2020 [79] observed that activated microglia have the capacity to release proinflammatory mediators leading to neuroinflammation. However, uncontrolled neuroinflammation can give rise to various neurological disorders including AD. Statins can inhibit neuroinflammation and associated neurodegenerative complications through inhibition of microglia activation and release of pro-inflammatory cytokines [79].

Nevertheless, many reported cases raised concern regarding cognitive adverse effects from the use of statins. Statins-induced memory loss from many case reports was conducted by self-reporting, which may be subjected to intra-individual and inter-individual variations [59].

About 50% of patients on atorvastatin and simvastatin therapy develop cognitive dysfunction within two months. This cognitive deficit was resolved upon discontinuation of statins therapy [59].

### 3.2. Beneficial Effects of Statins on AD

It has been shown that statins pharmacotherapy could be beneficial for AD patients mainly in dyslipidemic patients with homozygous ApoE4 [80,81]. Different studies illustrated that incidence and propagation of AD were low in statins-treated patients [82,83]. The protective effect of statins against the propagation of AD is mainly related to the effect on the ApoE4 allele which is linked with high circulating cholesterol levels. Expression of the ApoE4 allele and hypercholesterolemia are associated with a greater risk for the development of AD [84]. It has been shown that 95% of late-onset AD patients encoding ApoE4 as dysregulation of brain cholesterol metabolism is associated with the pathogenesis of AD [84]. Herein, statins pharmacotherapy could be effective in reducing the pathogenesis of AD through modulation of brain ApoE4. Of interest, early treatment with statins could be an effective therapeutic option for the prevention of AD, but this may be less effective at the onset of AD [85].

Remarkably, brain pathological changes start about 15–20 years before the onset of clinical symptoms of AD. Therefore, early prevention in the preclinical phase of AD is of great importance to attenuate the progression of this disease [86]. Re-analysis of data from the clinical trials examining the protective role of simvastatin on cognitive function in AD patients showed that this drug was mainly effective in AD patients with higher expression of ApoE4 [80]. Different studies indicated that statin users had superior cognitive scores compared to non-users, which were more evident with lipophilic statins [87,88]. An experimental study demonstrated that lovastatin cannot reduce brain cholesterol in ApoE4 knockout mice [89]. A preclinical study supports that statins effects may be independent from brain HMG-CoA reductase through anti-inflammatory and antioxidant effects [90]. In addition, an experimental study illustrated that administration of statins in mice reduced follitin, a raft biomarker in the neuronal membrane, through modulation of membrane cholesterol [91].

Many clinical studies observed the protective effects of statins against the development of cognitive impairments in AD patients. A population-based study from Taiwan involving 719 mild–moderate AD patients on statins pharmacotherapy revealed that early-use statins were associated with a significant reduction in the progression of AD [92]. Similarly, a Rotterdam study conducted by Haag et al. [83] found that statins therapy was associated with attenuation for progression of AD regardless of their lipophilicity. A prospective study followed 6992 patients on, or not on, various statins therapy from 1993 till 2005 for the incident of AD. During the study, 582 patients who were not on statins developed AD, whereas nonstatins also developed AD [83]. This finding suggests that the protective effect of statins against the progression of AD could be independent of statins’ lipophilicity. In addition, a cross-sectional study showed that statins have protection against the propagation of AD [93]. A recent systematic review and meta-analysis illustrated that statins therapy has no harmful effects on the neurocognitive function in AD patients and could be beneficial, mainly high-potency statins, against AD [94]. A meta-analysis conducted by Xuan et al. observed that statins therapy was linked with beneficial effects on AD patients [95]. Remarkably, a longitudinal study that followed patients with traumatic brain injury for 18.5 years revealed that statins alone, or in combination with angiotensin-converting enzyme inhibitors, prevent the development of AD and dementia with a history of traumatic brain injury [96]. Furthermore, an observational study and meta-analysis revealed that statins used for one year were associated with a reduction in the development of AD by about 20% in a dose-dependent manner [97]. As well, a systematic review and meta-analysis performed by Chu et al. [98] illustrated that statins pharmacotherapies reduce the risk of all types of dementia, mild cognitive dysfunction, and AD, but not vascular dementia. In addition, meta-analysis and the observational study revealed the protective effects of statins on the development of post-stroke dementia [99]. As well, statins used in combination with psychotropic agents have the ability to decrease inflammation-induced cognitive impairments and AD [100].

The underlying mechanisms of the protection of statins in AD are related to the inhibition of brain HMG-CoA reductase. Of note, polymorphism of HMG-CoA reductase and higher expression of ApoE4 enhance the pathogenesis of AD [101]. The association between hypercholesterolemia and the development of AD could be due to the deposition of cholesterol in the hippocampus with induction formation of amyloid precursors and progression of AD pathogenesis [102]. Statins inhibit Aβ formation through inhibition of brain cholesterol biosynthesis [103]. Epidemiological data confirmed that hypercholesterolemia and hyperlipoproteinemia, mainly ApoE, in midlife increase the risk for the development of late-onset AD [103]. Furthermore, depletion of cholesterol product isoprene affects neuronal stability and neurotransmission transduction [102]. Statins improve brain cholesterol homeostasis and isoprene concentration, which enhance neurotransmission transduction [97]. Notoriously, statins inhibit the synthesis and secretion of ApoE from microglia and astrocytes. It has been shown that ApoE is associated with the deposition of Aβ amyloid and the creation of senile plaques [79,104].

**Figure 6:**
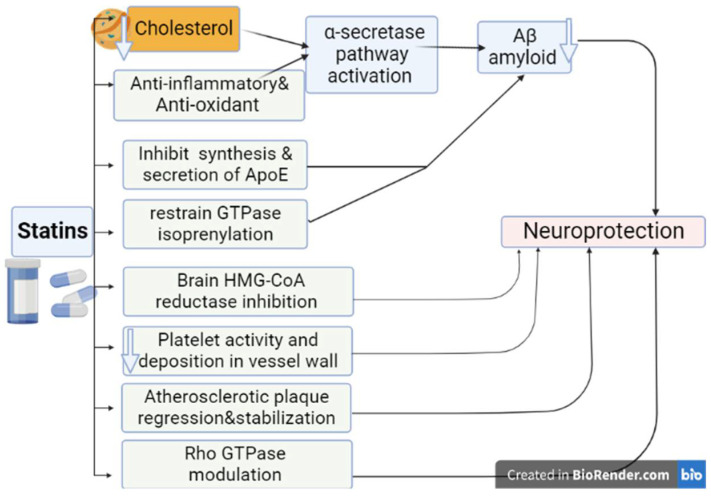
Beneficial effects of statins on Alzheimer’s disease (AD): Statins’ beneficial effects against development and progression of AD are mediated by different mechanisms including anti-inflammatory and antioxidant effects, modulation of Rho GPTase, inhibition synthesis and secretion of ApoE, and activation of α secretase pathway with reduced deposition of Aβ.

Moreover, statins promote cerebral blood flow (CBF) through the improvement of endothelial function and the release of nitric oxide (NO) [105]. As well, statins, in virtue of their anti-inflammatory and antioxidant effects with induction of α-secretase, inhibit the formation and deposition of Aβ [106]. It has been shown that α-secretase plays a critical role in the degradation of Aβ amyloid, preventing the amyloid formation and hippocampal injury in AD [107]. Thus, activation of α-secretase activity by statins could have a possible protective effect in preventing the development of AD [106,107]. In addition, statins attenuate brain ischemic reperfusion injury through modulation of Rho GTPase [108]. Interestingly, Rho GTPase is implicated in the pathogenesis of AD [109]. Moreover, statins such as simvastatin and lovastatin inhibit the production of amyloid precursors and reduce them in both cerebrospinal fluid and brain tissues, both in vivo and in vitro [110]. Statins selectively restrain GTPase isoprenylation in a dose-dependent manner with subsequent inhibition of Aβ amyloid [111]. This finding suggests that statins could be effective in the management of AD through GTPase isoprenylation-dependent pathway. Notably, low cholesterol concentration activates the α-secretase pathway with the reduction of Aβ amyloid formation [112]. Many longitudinal studies showed that hypercholesterolemia predicts the development of AD (Figure 6) [112,113].

Furthermore, statins’ effects on cognitive function may be related to ethnicity. It has been reported that statins’ effects on cognitive deficits were lower in the Mexican population but higher in the African population [114,115].

### 3.3. Neutral Effects of Statins on AD

Notably, many studies were carried out to find the positive and negative impacts of statins pharmacotherapy on the incidence of AD depending on the molecular relationship between Aβ amyloid formation and cholesterol level [116,117]. Nevertheless, these studies failed to find the significant therapeutic effect of statins pharmacotherapy on the incidence of AD [25,118]. As well, various randomized clinical trials revealed insignificant improvement effects of statins on cognitive function in AD patients. For example, simvastatin pharmacotherapy in 406 mild–moderate AD patients for an 18-month duration failed to produce any protective effect [119]. A randomized, placebo-controlled clinical trial conducted by Feldmen et al. in 2007 demonstrated that atorvastatin therapy for 72 weeks in 640 mild–moderate AD patients did not positively affect cognitive function [118]. A systematic review and meta-analysis involving 25 randomized controlled clinical trials of 46,836 statin users illustrated that statins therapy was not associated with cognitive dysfunction [120].

Many clinical trials and their reevaluation found no risk for AD and cognitive dysfunction from the use of statins [50,52]. However, these studies evaluate the cardiovascular outcomes rather than the cognitive outcomes regarding prolonged use of statins. As well, the design of these studies may limit the detection of cognitive dysfunction in patients with cardiovascular diseases [50,52]. Notably, a population-based study from the Mayo Clinic Study of Aging involving 1160 patients for assessment of neuroimaging biomarkers in AD patients aged more than 65 years with a history of cardiovascular complications on statins pharmacotherapy showed no significant correlation between long-term statins and tau/amyloid burden [121,122]. This finding proposed that statins pharmacotherapy had neutral effects on AD pathogenesis. An elegant systematic review involved 304 published articles disclosed that long-term statins therapy has not observed an improvement in cognitive function and did not lead to any significant amelioration in AD patients [123].

Furthermore, a prospective study on the effect of pravastatin on cognitive function in patients with cardiovascular disorders revealed no significant effect of pravastatin on cognition compared to placebo [124]. Similarly, simvastatin did not produce a significant effect on cognition compared to placebo [50]. Of interest, many systematic reviews and meta-analysis studies confirmed the neutral role of statins pharmacotherapy on cognitive function [51,52]. Therefore, statins pharmacotherapy seems not to be associated with a negative or positive impact on the cognitive function and development of AD.

The net effects of statins on the cognitive function and pathogenesis of AD regarding positive, negative, and neutral impacts is summarized in Table 1.

## 4. Conclusions

Alzheimer’s disease (AD) was used to describe pre-senile dementia in the younger age group. AD is characterized by the deposition of Aβ plaque and NFTs in the brain. The neuropathological changes in AD are related to the deposition of amyloid plaques, NFTs, and progression of neuroinflammation, neuronal mitochondrial dysfunction, autophagy dysfunction, and cholinergic synaptic dysfunction. Statins are one of the main cornerstone drugs for the management of cardiovascular disorders regardless of dyslipidemia status. Widespread and prolonged use of statins may affect the cognitive functions in healthy subjects and dementia patients. Statins-induced cognitive impairments in both patients and health providers had been reported according to the post-marketing survey. However, many patients with underlying cardiovascular disorders on statins pharmacotherapy experience some aspects of cognitive impairments. Evidence from prospective and observational studies gives no robust conclusion regarding the beneficial or detrimental effects of statins on the cognitive functions in AD patients, so further studies are warranted. The beneficial effects of statins are related to the selective restraint of GTPase isoprenylation in a dose-dependent manner with subsequent inhibition of Aβ amyloid. This finding suggests that statins could be effective in the management of AD through the GTPase isoprenylation-dependent pathway. Notably, low cholesterol concentration activates the α-secretase pathway with reduction of Aβ amyloid formation. Nevertheless, many reported cases raised concerns regarding cognitive adverse effects from the use of statins. Statins-induced memory loss from many case reports was conducted by self-reporting, which may be subjected to intra-individual and inter-individual variations. The potential link between the positive–negative impacts of statins pharmacotherapy on the incidence and progression of AD remain tentative requiring extensive research, including whether statins pharmacotherapy increases or decreases the progression of AD. Therefore, statins pharmacotherapy seems to be associated with a negative or positive impact on the cognitive function and development of AD. Taken together, according to the assorted view of preponderance, statins produce various effects including positive, negative, and neutral impacts on the cognitive function and the pathogenesis of AD. There are contradictions in the results and harmful effects cannot be ruled out; further investigation is needed. Again, there are no solid results to rule out the harm of the statins.

Therefore, statins therapy may continue in AD patients with cardiometabolic disorders without potential harm. Nevertheless, this narrative review cannot give the final conclusion regarding the precise effects of statins on AD neuropathology as the duration of statins therapy and the dose–response curve were not evaluated in clinical studies. Thus, experimental, clinical trials, retrospective, and large-scale prospective studies are recommended in this regard.

## Figures and Tables

**Table 1:** Effects of statins on the cognitive function and pathogenesis of AD.

Ref.	Study Type	Findings
Bitzur [24]	Observational study	Prolonged use of statins may affect the cognitive functions in healthy subjects and dementia patients.
Schultz et al. [49]	Narrative review study	Statins induced cognitive impairments in both patients and health providers.
Power et al. [51]	Randomized controlled trials and observational studies	Statins use does not support a causal preventative effect on cognitive decline or dementia.
McGuinness et al. [52]	Cochrane review	Statins use in late life of individuals at risk of vascular disease has no effect in preventing AD or dementia.
Posvar et al. [58]	Clinical trial	Atorvastatin in phase one clinical trial led to reversible cognitive impairment.
Muldoon et al. [60]	A randomized, double-blind clinical trial	Lovastatin leads to significant impairment of psychomotor performance and working memory compared to the placebo effect.
Evans et al. [61]	Patient survey-based analysis	Discontinuation of statins reverses cognitive impairment.
Sahebzamani et al. [63], Li et al. [64]	Observational study and review study	Lipophilic statins are associated with a higher percentage of reversible cognitive dysfunction.
Stuart et al. [66]	Experimental study	Hydrophilic pravastatin leads to more cognitive impairment compared to lipophilic atorvastatin in adult rats.
Zhang et al. [67]	A population-based, retrospective cohort study	Lipophilic and fungus-derived statins are associated with a higher risk for progression of AD compared to hydrophilic statins.
Strandberg et al. [78]	A preclinical study	Use of lovastatin promotes the formation and deposition of Aβ amyloid in female mice.
Wagstaff et al. [59]	A case reported study	About 50% of patients on atorvastatin and simvastatin therapy develop cognitive dysfunction within two months.
Jick et al. [82]	A case-control study	AD was low in statins-treated patients.
Sierra et al. [87], Eckert et al. [88]	A comparative study, a prospective clinical trial	Different studies indicated that statin users had superior cognitive scores compared to the non-users which were more evident with lipophilic statins [87,88].
Lin et al. [92]	A population-based study	Early use statins are associated with a significant reduction in the progression of AD.
Haag et al. [83]	A prospective study	Statins have the protective effects of statins against the progression of AD independent of lipophilicity.
Rockwood [93]	A cross-sectional study	Statins have protection against the propagation of AD.
Olmastroni et al. [94]	A systematic review and meta-analysis	Statins therapy has no harmful effects on the neurocognitive function in AD patients.
Xuan et al. [95]	A meta-analysis	Statins therapy has beneficial effects on AD patients.
Wood et al. [97]	Observational study and meta-analysis	Statins use is associated with a reduction risk of AD development by about 20% in a dose-dependent manner.
Chu et al. [98]	A systematic review and meta-analysis	Statins pharmacotherapy reduces the risk of all types of dementia, mild cognitive dysfunction, and AD, but not vascular dementia.
Yang et al. [99]	A meta-analysis and observational study	Statins have the protective effects against the development of post-stroke dementia.
Sano et al. [119]	A randomized, double-blind, placebo-controlled trial	Simvastatin in mild–moderate AD patients for an 18-month duration failed to produce any protective effect.
Feldman et al., 2010 [118]	A randomized, placebo-controlled clinical trial	Atorvastatin therapy for 72 weeks in mild–moderate AD patients did not positively affect cognitive function.
Ott et al., 2015 [120]	A systematic review and meta-analysis	Statins therapy was not associated with cognitive dysfunction.
Ramanan et al., 2018 [121]	A population-based study	No significant correlation between long-term statins and tau/amyloid burden.
Mejías-Trueba et al., 2018 [123]	A systematic review	Long-term statins do not have any improvement on the cognitive function and did not lead to any significant amelioration in AD patients.
Shepherd et al., 1999 [123]	A prospective study	The effect of pravastatin on cognitive function in patients with cardiovascular disorders revealed no significant effect on the cognition compared to placebo.

## Data Availability

Not applicable.
